# Pharmacovigilance systems in resource-limited settings: an evaluative case study of Sierra Leone

**DOI:** 10.1186/s40545-019-0173-2

**Published:** 2019-06-11

**Authors:** Onome T. Abiri, Wiltshire C. N. Johnson

**Affiliations:** 1 Department of Pharmacology, Faculty of Basic Medical Sciences, College of Medicine and Allied Health Sciences, University of Sierra Leone, Freetown, Sierra Leone; 2 Department of Clinical Pharmacy and Therapeutics, Faculty of Pharmaceutical Sciences, College of Medicine and Allied Health Sciences, University of Sierra Leone, Freetown, Sierra Leone; 3 Pharmacy Board of Sierra Leone, Freetown, Sierra Leone

**Keywords:** Pharmacovigilance systems, Indicators, Public health programmes, Health facilities

## Abstract

Few low and middle-income countries (LMIC) have fully operational pharmacovigilance structures, systems and legal framework to collect and collate safety data and evaluate the risks and benefits by active and passive approaches. However, in a LMIC such as Sierra Leone, the capacity to manage the risks by taking appropriate preventative actions to help inform therapeutic decisions, promote rational use of medicines, guide risk management and communications is gradually growing but yet to be fully optimized.

This study sought to assess the current status of pharmacovigilance in Sierra Leone since it became the 87th member of the World Health Organisation International Drug Monitoring Programme. This study evaluated the pharmacovigilance system in Sierra Leone through a comprehensive and system-based approach that covered the national medicines regulatory authority, health facilities and public health programmes. A descriptive cross-sectional study design was employed. Using a convenience sampling method, 14 respondents from the national medicines regulatory authority, six health facilities and six public health programmes were interviewed.

Data were collected using a validated metric instrument: Indicator-Based Pharmacovigilance Assessment Tool. A scoring system was used for the quantification of assessment results with a score greater than 60% indicating that an organization has structural and policy frameworks to collect and collate safety data in a national database and evaluate the risks and benefits by both active and passive approaches.

The study findings showed that the national medicines regulatory authority scored 79% and thus met the standard requirements of pharmacovigilance. On the other hand, the health facilities and public health programmes scored less than 60% indicating the need to fully operationalise pharmacovigilance frameworks at these levels. The study further demonstrated that the national medicine regulatory authority which hosts the national pharmacovigilance centre had functional pharmacovigilance structures and processes with potential to providing leadership in the implementation of pharmacovigilance in Sierra Leone.

**Electronic supplementary material:**

The online version of this article (10.1186/s40545-019-0173-2) contains supplementary material, which is available to authorized users.

## Introduction

The definition and scope of pharmacovigilance (PV) has evolved over the years to accommodate systems approach for improving the safe use of medicines. The World Health Organisation (WHO) has defined pharmacovigilance as the science and activities relating to the detection, assessment, understanding and prevention of adverse effects or any other medicine-related problems [1]. This definition covers all medicines including allopathic, complementary and alternative, biotherapeutics and vaccines.

A comprehensive PV system involves more than just risk identification and data collection. It also takes into consideration risk evaluation, minimisation and communication. Such systems protect the public from medicine-related harms through efficient and timely detection, reporting, assessment, communication and prevention of medicine-related adverse events through people and structures that have the authority to take appropriate action [2].

Some local and international institutions are playing key roles in strengthening PV to guarantee medicines safety in resource-limited settings through funding mechanisms such as the US President’s Emergency Plan for AIDS Relief (PEPFAR), Global Fund (GF) to fight AIDS, Tuberculosis and Malaria, the US President’s Malaria Initiative (PMI) and Global Alliance for Vaccines and Immunisation (GAVI). Several technical agencies, like the WHO and its PV collaborating centres in Uppsala, Rabat, Lareb, Accra and India and the United States Pharmacopeia/Promoting the Quality of Medicines (USP/PQM) programme are also providing financial and capacity building support to build PV systems in settings were resources are scarce [2].

Funding support and initiatives by these international organisations in resource-limited countries such as Sierra Leone have resulted in increased access to medicines and vaccines for the management of public health diseases. The number of artemisinin-based combination therapy (ACT) courses procured for African countries for treatment of malaria increased from 11.2 million in 2005 to 158 million in 2009. Similarly, about four million people had access to antiretroviral therapy in sub-Saharan Africa (SSA) in 2009, compared to only 50,000 in 2002 [3]. The Global Fund alone has committed US$ 21.9 billion with 37% of funding allocated for health commodities [4].

With increased access to newly introduced essential medicines and vaccines such as novel Ebola vaccine that would be introduced and deployed in Sierra Leone for the first time on a large scale, there is a greater need to monitor and promote their safety and effectiveness through an effective PV system. Despite extensive studies and usage of many medicines and vaccines in developed countries, their safety profiles may not be the same in other settings, given that the pattern, severity and incidence of adverse drug reaction (ADRs) may vary because of genetic and local environmental factors.

It is therefore critical that medicines continue to be monitored for safety and effectiveness as soon as they are introduced into the market under real-life conditions. For some medicines, problems will only occur in real-world situations following prolonged use, usage in specific sub-populations or in patients with various co-morbidities that are not endemic in developed countries where clinical studies were conducted and/or the products have been used [5].

The Pharmacy Board of Sierra Leone (PBSL) which is the National Medicines Regulatory Authority (NMRA), also houses the National Pharmacovigilance Centre in Sierra Leone. As a full member of the WHO International Drug Monitoring Programme, the PBSL promotes and supports PV in health facilities, public health programmes, universities and research institutions through the establishment of effective medicines monitoring system.

An effective PV system protects the public through efficient and prompt recognition, collection, recording and evaluation of adverse events and by communicating benefits and risks to corroborate therapeutic decision-making at different strata of the health care system. Few low and middle-income countries have fully operational PV structures, systems and legal frameworks to collect and collate safety data and evaluate the risks and benefits by both passive and active approaches. However, in countries such as Sierra Leone, the capacity to manage the risks by taking appropriate preventive actions to help inform therapeutic decisions, promote rational use of medicines and guide risk management and communication is gradually growing but yet to be fully optimised.

Of 46 SSA countries studied by the Strengthening Pharmacovigilance Systems (SPS) programme, 87% did not have a functional PV system, 59% did not have a national policy related to medicine safety, 70% lacked legislation to monitor adverse events, 26% did not have a national PV center, and 61% lacked a medicine safety advisory committee. Furthermore, 74% was reported to have spontaneous adverse event reporting systems, while less than 50% monitored product quality, medication errors, or treatment failures [6]. A similar study conducted in five Asian countries namely Bangladesh, Cambodia, Nepal, Thailand and the Philippines indicated that amongst the 86 health facilities evaluated, less than 50% of them had a PV center or unit, or designated staff for PV-related activities within their facilities. Fifteen percent had Drug and Therapeutics Committee (DTCs) and 38% had Drug Information Services (DIS), while for the 19 Public Health Programmes (PHPs) assessed, 26% were reported to have dedicated funds available, 53% had Standard Operating Procedures (SOPs) and 58% had guidelines in place that addressed elements of PV [7].

Since no PV assessment has been done in Sierra Leone from the time it became the 87th member of the WHO International Drug Monitoring programme in 2008, this study therefore aimed to evaluate the current status of PV in Sierra Leone through a comprehensive and system-based approach that covered the PBSL, healthcare facilities and PHPs.

## Methods

### Study setting

Sierra Leone is a country in West Africa that is bordered by Guinea to the northeast, Liberia to the southeast, and the Atlantic Ocean to the southwest. The country covers a total area of 71,740 km^2^ (27,699 sq. mile) and with an estimated population of 7 million [8]. Sierra Leone is divided into five administrative regions: the Northern Region, North-Western Region, Eastern Region, Southern Region and the Western Area; which are subdivided into 16 districts. The national public health system of Sierra Leone consists of 986 peripheral health units, 53 secondary health facilities and a teaching hospital complex that consist of six tertiary referral teaching hospitals (8). The ratios of physician and pharmacist to patient population is 2:50,000 and 1: 50,000 respectively [9, 10].

### Study design and sampling

This was a descriptive cross-sectional study that was conducted in Sierra Leone from April to September 2016. Fourteen respondents recruited by convenience sampling method from the PBSL, six health facilities and six PHPs were interviewed. The study participants consisted of two pharmacists from PBSL, three pharmacists and three medical doctors from the health facilities and six pharmacists from the PHPs. Study participants were recruited from the following institutions:

- The PBSL
- Six health facilities
  - ○Three teaching hospitals located in the capital city, Freetown:
    - ▪ Connaught Teaching Hospital (CTH)
    - ▪ Ola During Children Hospital (ODCH)
    - ▪ Princess Christian Maternity Hospital (PCMH)
  - ○Three regional referral hospitals located in the southern, eastern and northern regions respectively):
    - ▪ Bo government hospital (BoGH)
    - ▪ Kenema government hospital (KeGH)
    - ▪ Makeni government hospital (MaGH)
- Six Public Health Programmes (PHPs):
  - ○The National Malaria Control Programme (NMCP)
  - ○National Tuberculosis and Leprosy Control Programme (NLTCP)
  - ○The HIV/AIDS Control Programme (HIV/AIDS)
  - ○The Reproductive and Child Health Programme (RCH)
  - ○The Expanded Programme on Immunisation (EPI)
  - ○The Neglected Tropical Disease Control Programme (NTDCP)

### Data collection

#### Data collection tool

The Indicator-based Pharmacovigilance Assessment Tool (IPAT) (2) was used for data collection. The tool was designed and validated by the Management Sciences for Health, a US-based not-for--profit organization through its strengthening of pharmaceutical systems programme specifically for the assessment of pharmacovigilance systems in developing countries.

IPAT has 43 indicators including 26 core and 17 supplementary indicators. These indicators cover five pharmacovigilance medicine safety monitoring components. The purpose of the tool is to enable the conduct of pharmacovigilance assessment through a series of questions focusing on structures, processes and impact of pharmacovigilance systems. The five areas of medicine safety monitoring covered are:

1. Policy, law and regulation (4 indicators, 1.1–1.4)
2. Systems, structures and stakeholder coordination (15 indicators, 2.1–2.15)
3. Signal generation and data management (6 indicators, 3.1–3.6)
4. Risk assessment and evaluation (8 indicators, 4.1–4.8)
5. Risk management and communication (10 indicators, 5.1–5.10)

#### Data collection level

The first area (policy, law and regulation) represents data collected at the national level such as the NMRA. The other four areas are important for data collection for hospitals and public health programmes.

In this study, the indicators were classified according to their relevance to health system levels and where they could be collected:

- 39 indicators for NMRAs (1.1–1.4, 2.1–2.15, 3.1–3.6, 4.1–4.5, 5.1–5.7 and 5.9–5.10)
- 21 indicators for hospitals (2.1–2.5, 2.8–2.11, 2.13, 3.3–3.6, 4.1, 4.3–4.5, 5.1 and 5.3–5.4)
- 25 indicators for PHPs (1.1, 2.1–2.2, 2.4–2.5, 2.8–2.9, 2.13, 3.3–3.6, 4.1–4.8, 5.1–5.3, 5.7 and 5.9)

#### Data collection process

Prior to data collection, the participants were requested to take part in the study through face to face contacts, emails and telephone calls. The respondents provided information with respect to the indicators on the IPAT tool administered to them through face-face interviews from April to June 2016. Relevant documents on PV were also collected from the respondents and reviewed by the investigators. These documents served as evidence in support of the interviews. For the NMRA, documents reviewed were National Medicines Policy 2012, Pharmacy and Drugs Act 2001, revised Pharmacy and Drugs Act 2012, 2015 annual report, 2015 regulatory and medicines information bulletins, pharmacovigilance guidelines. Documents reviewed from the health facilities included 2015 annual reports, job descriptions and SOPs of PV focal persons and treatment guidelines. The PHPs documents reviewed covered treatment guidelines, policies, SOPs and 2015 annual reports.

### Data analysis

Analysis of data was quantitative and qualitative and Microsoft Excel (Microsoft Corporation, Redmond, WA, USA) was used to compute the scores. With respect to scoring of the indicators, a core indicator recorded as ‘Yes’ was allocated a score of 2; a supplementary indicator recorded as ‘Yes’ was allocated a score of 1; any core or supplementary indicator recorded as ‘No’ was given a score of 0. These numerical values assigned are in line with the IPAT tool scoring system. Recommended thresholds were indicated by the IPAT manual for the quantitative indicators, i.e. indicators with numbers and percentages (2.13, 4.4, 5.3, 5.4, 5.5, 5.6, 5.7 and 5.10), resulting in a total score of 52 for core indicators and 17 for the supplementary indicators. The score attained by the indicators was divided by the total score of all the indicators and multiplied by 100. If this value is greater than 60% for each component of health system level, the component is said to meet the standard PV requirements for that health system level (2, 6). Results were presented as tables and were used to compare performance of indicators with the same component.

### Ethical considerations

Authorisation to conduct the study was obtained from the relevant institutions where the study was done, that is, the Pharmacy Board of Sierra Leone, the PHPs and hospitals. The participants gave their consent to take part in the study before they were interviewed. Data were processed anonymously to ensure confidentiality.

## Results

Fourteen respondents from 13 institutions including two respondents from PBSL, six from health facilities and six from PHPs were interviewed. The sample size consisted of 11 pharmacists and 3 medical doctors.

### Pharmacovigilance performance of PBSL

Thirty-nine indicators (23 core and 16 supplementary) and (19 structure, 11 process and 9 outcome) were employed to assess the PBSL for a maximum score of 62 (Additional file 1: Table S1. Result for the Pharmacy Board of Sierra Leone).

Pharmacy Board of Sierra Leone got an overall score of 79% (49/62). The performance by pharmacovigilance (PV) area is as follows (Table 1):

**Table 1:** Pharmacovigilance performance of PBSL

PV component	Score (%)	Target outcome
Policy, law and regulation	33 (2/6)	Not achieved
Systems, structures and stakeholders coordination	96 (25/26)	Achieved
Signal generation and management	100 (12/12)	Achieved
Risk assessment and evaluation	43 (3/7)	Not achieved
Risk management and communication	64 (7/11)	Achieved
Overall score	79 (49/62)	Achieved

The findings of the study revealed that the required policy statements for policy, law, and regulation for PV in Sierra Leone was in place but no specific legislation for PV was available. The National Medicines Policy (NMP) recognized the need for PV and mandated PBSL with the responsibility for pharmacovigilance and clinical trial regulation [11].

However, specific legal provision for the enforcement of PV is currently not available in Sierra Leone as the current Sierra Leone Pharmacy and Drugs Act 2001 does not include pharmacovigilance. The Pharmacy and Drugs Act also did not place responsibility on marketing authorisation holders (MAHs) for product stewardships with respect to mandatory reporting of all serious adverse events (SAEs) or suspected unexpected serious adverse reaction (SUSARs) to the NMRA and conduct of post-marketing surveillance activities (Table 2).

**Table 2:** Policy, law and regulation

Policy, law and regulation	Document Availability
PV policy	Available
PV legislation	Not available
Legal provision for MAHs to report ADRs	Not available
Legal provision for MAHs to conduct post-marketing surveillance activities	Not available

Consequently, the Pharmacy and Drugs Act has been revised by the NMRA and presented for ratification by the parliament. PBSL has also developed guidelines to implement a system of Qualified Person for Pharmacovigilance (QPPV) that will mandate and ensure that MAHs have functional pharmacovigilance systems in place so that they can assume responsibility and liability for their products in the market and to ensure that appropriate actions are taken when necessary [12].

### Pharmacovigilance performance of health facilities

Twenty-two indicators (15 core and 7 supplementary) and (10 structure, 8 process and 4 outcome) were employed to assess the health facilities for a maximum score of 37 (Additional file 1: Table S2. Result for health facilities).

The overall scores for the health facilities were (Table 3):

**Table 3:** Pharmacovigilance performance health facilities

Health facilities	Score (%)	Target outcome
Connaught Teaching Hospital	51 (19/37)	Not achieved
Princess Christian Maternity Hospital	51 (19/37)	Not achieved
Ola During Children Hospital	22 (8/37)	Not achieved
Bo Government Hospital	54 (20/37)	Not achieved
Kenema Government Hospital	54 (20/37)	Not achieved
Makeni Government Hospital	54 (20/37)	Not achieved

### Pharmacovigilance performance of public health programmes (PHPs)

Twenty-five indicators (16 core and 9 supplementary) and (8 structure, 12 process and 5 outcome) were employed to assess the PHPs for a maximum score of 41 (Additional file 1: Table S3. Results for the public health programmes).

The overall scores for the PHPs were (Table 4):

**Table 4:** Pharmacovigilance performance of public health programmes

Public health programmes	Score (%)	Target outcome
National Malaria Control Programme	54 (22/41)	Not achieved
Neglected Tropical Diseases Control Programme	51 (21/41)	Not achieved
Expanded Programme on Immunisation	44 (18/41)	Not achieved
HIV/AIDS Control Programme	37 (15/41)	Not achieved
National Leprosy and Tuberculosis Control Programme	39 (16/41)	Not achieved
Reproductive and Child Health Programme	34 (14/41)	Not achieved

Of the PHPs assessed, only 2 of 6 (33%) i.e. neglected diseases and immunisation programmes reported having a policy document containing essential statements on PV.

## Discussion

The national PV centre of the NMRA, Pharmacy Board of Sierra Leone scored 79% and met the standard requirements of PV by scoring greater than 60% while none of the health facilities and PHPs satisfied these criteria.

The lack of relevant PV legislations and regulations in Sierra Leone reflects fundamental limitation for enforcing medicine safety monitoring. This omission limits the capacity PBSL to mandate post-marketing safety commitments of the MAHs. Existence of a legislation, guidelines and policies having relevant statements on PV shows that a country has demonstrated high-level commitment to improve medicine safety and hence provide direction to enhance the system. The existence of legislations and regulations also support a strong legal foundation that ensures conformity by relevant stakeholders.

A similar study done in sub-Saharan Africa (SSA) that included 46 countries, showed that 19 countries (41%) have a national policy related to PV; 14 countries (30%) provided a legal mandate to monitor medicine-related adverse events. Only 13 countries (28%) have legal provisions that require MAHs to report all serious ADRs to the NMRA and eight countries (17%) require MAHs to conduct post-marketing surveillance activities [6].

Systems, structures, and stakeholder coordination have grown over the years with respect to PV in Sierra Leone. Sierra Leone has established basic structures for conducting PV activities, including a PV centre with clear mandate, defined roles and responsibilities, formal organizational structure, designated persons for pharmacovigilance, functional information and communication technology infrastructure, a national PV guideline, a medicine safety advisory committee and collaboration with the Uppsala Monitoring Centre (UMC) which is the World Health Organisation (WHO) Collaborating Center for International Drug Monitoring in Uppsala, Sweden. Through this collaboration, Sierra Leone became the 87th member of the WHO programme in 2008.

However, across the health facilities and public health programmes, lack of a dedicated budget for PV, pharmacovigilance training for health care workers, standard operating procedures (SOPs) and guidelines for PV-related activities, drug information services (DIS) and drug and therapeutics committees (DTCs) performing PV-related activities was reported. Kabore and colleagues also published results compatible to this study [13].

The development of sustainable PV systems and their optimal functioning are critical for effective medicines safety monitoring. Some of the factors required for a sustainable PV system include political will, financial support from government and partners; and also, strong collaborations and cooperation of health care professionals and health institutions.

Structures, systems and roles provide a foundational basis for organised and systematic operationalisation of PV activities. This will enable effective and efficient use of staff, skills, and tools. The implications of the lack of PV guidelines in health facilities and PHPs can lead to the inability to map and streamline stakeholders’ contributions to PV. This paucity has also contributed to the poor functioning of PV structures and systems within the health facilities and PHPs in relation to implementing and advancing PV activities. The development and implementation of comprehensive guidelines and SOPs will serve as a basis for structured and coordinated PV activities and optimum performance by various stakeholders. The lack of dedicated PV budget, procedures and training in pharmacovigilance for healthcare professionals indicates the inability and lack of capacity to consistently address PV issues. Where there are no proper structures, systems, and clear roles and responsibilities, there will be no fundamental basis for organised and systematic operationalisation of pharmacovigilance activities.

For signal generation and data management, the national PV centre has ADR reporting forms for consumer reporting, mass medicines administration campaigns and health care professionals. The spontaneous reporting system covers the full scope of PV, including product quality, medication errors, and treatment failures that can be reported by using the current ADR form. The central database of the national PV centre contains data from various sources, such as reports from clinical trials, adverse event following immunisation (AEFI) from the EPI/Child Health Programme, and ADRs from PHPs such the Malaria Control programme, the Tuberculosis and Leprosy Control Programme, Neglected Tropical Disease Control Programme and health facilities. Serious adverse event (SAE) reports from clinical trials are sent to the national PV centre, hence, there is an opportunity to draw from the inherent advantage of linking pre-marketing phases I, II and III and post-marketing safety data. Such coordination of PV data from various sources will enhance the effective synthesis, interpretation, and use of safety information. However, low reporting rates from health facilities and PHPs to the PV centre can be a challenge for signal generation.

Signal detection through reporting of suspected adverse events is the first step in the PV process, followed by signal evaluation and risk management [14]. Generation of signals depends on well sensitised healthcare professionals who report suspected adverse events. With this poor reporting rate observed in this study of suspected adverse events such as treatment failure and poor product quality, one can conclude that the national PV centre has few reports in its database, which will make detection and generation of signals to address safety issues challenging.

In Sierra Leone, factors such as lack of awareness of healthcare professionals and resources to promote pharmacovigilance contribute to poor ADR reporting rates. Low reporting rates of ADRs have implications for the ability to generate and evaluate signals of public health significance. When such risk assessment efforts are not undertaken, opportunities to learn about the safety and effectiveness of medicines during real-life use are lost. Subsequently, opportunities to use such new knowledge to inform treatment decisions by clinicians and pharmacists are also not utilised.

In relation to risk assessment and evaluation, the study revealed that no medicine utilisation reviews, studies quantifying incidence of medication errors and active surveillance studies have been conducted over the past years. The periodic review of the number and types of medicine-related adverse events through passive surveillance reporting as well as evaluation of significant safety issues through active surveillance are fundamental attributes of any comprehensive pharmacovigilance system. With respect to the number of ADRs reported in the previous year, IPAT recommends the use of thresholds to determine whether the number of reported adverse events meets that expected for a minimally functional PV system. For example, using a threshold of 100 reports per million populations per year, Sierra Leone will be expected to generate about 700 reports per year, and a health facility with about 50,000 people in its catchments area will be expected to generate a minimum of 5 reports per year to meet the threshold. The number of ADR reports received in Sierra Leone for the year under review was 1084 according to the PBSL 2015 annual report [15].

However, 95% of these reports were obtained from mass medicine distribution campaigns with artesunate-amodiaquine and albendazole-ivermectin, which suggested that the reporting rate might drop in the coming years without such initiatives. A study conducted by the strengthening pharmaceutical systems programme revealed that reporting rate of adverse event was minimal in the 46 SSA countries that were surveyed; only two of the countries surveyed collected more than 100 reports per million populations in 2010 (i.e. Burkina Faso and Namibia), and most countries generated less than 20 reports per million populations per year [6].

Without collection, reporting and analysis of adverse events, signals of public health importance will be missed and opportunities to learn about the safety and effectiveness of medicines during real-life use will also be lost. This finding has consequences for the ability to generate signals and evaluate safety issues of public health importance. Acknowledging that PV is still relatively new to Sierra Leone and that the PV centre became a member of the WHO programme in 2008, considerable effort and time are required to raise awareness among health care professionals and consumers on the significance of reporting adverse events.

From the risk management and communication perspective, this study showed that the PV centre requires MAHs to have risk mitigation plans (RMP) for high-risk medicines so as to prevent and manage ADRs by averting serious known risks. In Sierra Leone, between 2015 and 2016, MAHs of products such as Tivicay (Dolutegravir) for HIV/AIDS, Orasure and Congenix Ebola diagnostics were required to submit risk management plan (RMP) to the NMRA and are been monitored on a quarterly basis to ensure compliance [15]. Requesting safety information, responding to safety information, using bulletins to publish and communicate safety information to health care workers and the community still require considerable work so as to bolster risk management and communication across all health institutions. Risk management and communication is a crucial aspect of PV with high impact in preventing harm from medicines use. The essential medicines list contains products such as the fluoroquinolones and antiretrovirals that require risk management, but no strategies are in place to ensure safe use of these products in health facilities and PHPs. These facilities do not have guidelines for managing high-risk medicines. This situation can immensely affect the quality of patient care. The application of medicines utilisation reviews, risk management, and risk communication can enable PV to contribute to the improvement of health outcomes.

This study has some limitations. Firstly, a small number of study participants recruited through convenience sampling was employed for this study. Other key PV stakeholders such as health professional bodies such as the Sierra Leone Medical and Dental Council, Nurses Board. and private sectors were not included. This could affect the generalisability of the results. Secondly, the study relied on respondents’ assertions unless in cases where documentations were available as a source of verification. Nevertheless, the findings of the study have potential for providing platform for future research agenda on PV implementation in Sierra Leone and similar settings.

## Conclusion

The study demonstrates that PBSL which hosts the national PV centre, had the basic PV structures and processes in place and showed potential to providing leadership in implementation of PV in Sierra Leone. However, the study uncovered some gaps, mainly related to the lack of pharmacovigilance-specific legislation. The Pharmacy Board of Sierra Leone lacks the regulatory framework mandated by law and capacity for enforcement in order to ensure that industries assume responsibilities and liabilities for their products in the market and take appropriate actions when necessary.

Health facilities and PHPs have PV systems and structures that are currently weak and the ability to generate signals, evaluate them and use the information for risk management and communication is limited. Incorporation of both active and passive approaches with careful strategic planning can improve the impact of PV and consequently, improve patient safety. A great challenge and opportunity exist to improve the systems and capacities required to assure patient safety and to improve health outcomes in Sierra Leone.

Therefore, PV legislation should be in place to adequately address medicines safety monitoring nationwide and this should be backed with sufficient and dedicated budget. Active surveillance activities such as registries, sentinel sites and cohort event monitoring should be incorporated into the national PV system through close collaborations with research institutions, universities and PHPs so as to increase ADR reporting and aid signal detection. The National PV Centre of PBSL should collaborate with health professional regulatory bodies such as the Sierra Leone Medical and Dental Council, the Pharmaceutical Society of Sierra Leone, the Nurses Board and the universities teaching health-related discipline to ensure locally relevant PV topics are included in pre- and in-service training programmes.

## Additional file

Additional file 1: **Table S1.** Result for the Pharmacy Board of Sierra Leone. **Table S2.** Result for health facilities. **Table S3.** Result for the public health programmes. (DOCX 40 kb)

## Abbreviations

ADR: Adverse Drug Reaction
IPAT: Indicator-based Pharmacovigilance Tool
MAHs: Marketing Authorisation Holders
NMRA: National Medicine Regulatory Authority
PBSL: Pharmacy Board of Sierra Leone
PHPs: Public Health Programmes, PV Pharmacovigilance
QPPV: Qualified Person for Pharmacovigilance
WHO: World Health Organisation
