# Supplementary material for: Pharmacovigilance systems in resource-limited settings: an evaluative case study of Sierra Leone
Source: J Pharm Policy Pract. 2019 Jun 11;12:13. doi: 10.1186/s40545-019-0173-2 (PMC6558671; doi:10.1186/s40545-019-0173-2)
Supplement: Supplementary file 1 — Table S1. Result for the Pharmacy Board of Sierra Leone. Table S2. Result for health facilities. Table S3. Result for the public health programmes. (DOCX 40 kb) [file 40545_2019_173_MOESM1_ESM.docx]

**Additional file 1**

**Table S1 Result for the Pharmacy Board of Sierra Leone**

|  | **Table 1 Result for the NMRA** |  |  |
| --- | --- | --- | --- |
|  | PV component | Type of indicator | Score |
|  | 1. **I. Policy, law and regulation** |  |  |
| 1.1(S) | Existence of a policy document that contains essential statements on pharmacovigilance(PV) | C | 2 |
| 1.2 (S) | Existence of specific legal provisions for pharmacovigilance in the medicines legislation | C | 0 |
| 1.3 (S) | Legal provision for the marketing authorization holder(MAH) mandatorily report serious adverse drug reaction (ADR) to the NMRA | S | 0 |
| 1.4 (S) | Legal provision for MAH to conduct post marketing safety activities | S | 0 |
|  | **Subtotal score (%)** |  | **2/6 (33)** |
|  | **II Systems, structures and stakeholders coordination** |  |  |
| 2.1 (S) | Existence of a PV centre | C | 2 |
| 2.2 (S) | Clear mandate, structure, roles, and responsibilities for the PV centre | C | 2 |
| 2.3 (S) | Existence of an information service that provides medicine safety–related question-and-answer services | C | 2 |
| 2.4 (S) | A designated staff responsible for PV | C | 2 |
| 2.5 (S) | Dedicated budget available for PV-related activities | C | 2 |
| 2.6 (S) | Existence of a national medicine safety advisory committee that has met at least once in the last year | C | 2 |
| 2.7 (S) | Existence of national PV guidelines | C | 2 |
| 2.8 (S) | Existence of standard operating procedures (SOPs) for improving patient safety relating to medicine use | C | 2 |
| 2.9 (S) | Existence of communication technologies to improve safety reporting and provision of medicine information | C | 2 |
| 2.10 (S) | Existence of a medicine safety bulletin or newsletter published in the last six months | C | 2 |
| 2.11 (S) | Existence of core reference materials available in the PV centre | S | 1 |
| 2.12 (S) | Core PV topics present in the preservice training curricula disaggregated by medicine, pharmacy, nursing, and public health curricula | S | 0 |
| 2.13 (S) | Health care providers trained on pharmacovigilance and medicine safety in the last year | S | 1 |
| 2.14 (S) | Existence of a platform for the coordination of pharmacovigilance activities at the national level | C | 2 |
| 2.15 (S) | Membership of the WHO Collaborating Centre for International Drug Monitoring (UMC) | S | 1 |
|  | **Subtotal score (%)** |  | **25/26 (96)** |
|  | **III Signal generation and management** |  |  |
| 3.1 (P) | Existence of a system for coordination and collation of pharmacovigilance data from all sources | C | 2 |
| 3.2 (P) | Existence of a database for tracking pharmacovigilance activities | C | 2 |
| 3.3 (P) | Existence of a form for reporting suspected ADRs | C | 2 |
| 3.4 (P) | Existence of a form for reporting suspected product quality issues | C | 2 |
| 3.5 (P) | Existence of a form for reporting suspected medication errors | C | 2 |
| 3.6 (P) | Existence of a form for reporting suspected treatment failure | C | 2 |
|  | **Subtotal score (%)** |  | **12/12 (100)** |
|  | **IV Risk assessment and evaluation** |  |  |
| 4.1 (P) | Medicine utilization reviews carried out in the last year | S | 0 |
| 4.2 (P) | Pharmaceutical product quality survey conducted within the last five years | S | 1 |
| 4.3 (P) | Quantification of the incidence of medication errors in the last year | S | 0 |
| 4.4 (P) | Number of ADR reports received in the last year | C | 2 |
| 4.5 (P) | Active surveillance activities currently ongoing or carried out in the last five years | C | 0 |
|  | **Subtotal score (%)** |  | **3/7 (43)** |
|  | **Table 1 Result for the…continued** |  |  |
|  | Pharmacovigilance component | Type of indicator | Score |
|  | **V. Risk management and communication** |  |  |
| 5.1 (O) | Risk mitigation plans currently in place that are targeted at high-risk medicines | S | 1 |
| 5.2 (O) | Prequalification schemes used in medicine procurement decisions | S | 1 |
| 5.3 (O) | Number of medicine safety information requests received and addressed in the last year | S | 0 |
| 5.4 (O) | Planned issues of the medicine safety published a newsletter or bulletin in the last year | S | 1 |
| 5.5 (O) | Number of medicine safety issues of local relevance identified from outside sources acted on locally in the last year | S | 1 |
| 5.6 (O) | Number of “Dear health care professional” letters or other safety alerts developed and distributed in the last year | S | 0 |
| 5.7 (O) | Average time lag between identification of safety signal of a serious ADR and communication to health care workers and the public | C | 0 |
| 5.9 (O) | Public or community education activities relating to medicine safety carried out in the last year | S | 1 |
| 5.10 (O) | Percentage of medicines sampled in the last year that passed product quality tests | C | 2 |
|  | **Subtotal score (%)** |  | **7/11 (64)** |
|  | **Total score achieved (%)** |  | **49/62 (79)** |

A grade of 2 points was allocated to each core indicator (C) fulﬁlled, 1 point for each supplementary indicator (S), and 0 points for any indicator not fulﬁlled. Thresholds set for quantitative indicators: 2.13 = 5 % of healthcare workers trained; 4.4 = 100 reports per million people; 5.3 = 100 per million people; 5.4 = at least 70 % of planned issues published; 5.5 = at least 70 % of relevant alerts have been acted on; 5.6 = at least 70 % of alerts communicated to healthcare professionals; 5.7 = at least 70 % of alerts communicated to healthcare professionals within 3 weeks; 5.10 = 80 % of sampled medicines pass the quality test. Outcome indicator (O), process indicator (P), structural indicator (S)

**Table S2: Result for health facilities**

|  | **Table 2 Result for health facilities** |  |  |  |  |  |  |  |
| --- | --- | --- | --- | --- | --- | --- | --- | --- |
|  |  |  |  |  |  |  |  |  |
|  | PV component | Type of indicator | CTH | PCMH | ODCH | BoGH | KeGH | MaGH |
|  | **II Systems, structures and stakeholders coordination** |  |  |  |  |  |  |  |
| 2.1(S) | Existence of a PV centre | C | 2 | 2 | 0 | 2 | 2 | 2 |
| 2.2(S) | Clear mandate, structure, roles, and responsibilities for the PV centre | C | 2 | 2 | 0 | 2 | 2 | 2 |
| 2.3(S) | Existence of an information service that provides medicine safety–related question-and-answer services | C | 0 | 0 | 0 | 0 | 0 | 0 |
| 2.4(S) | A designated staff responsible for PV | C | 2 | 2 | 0 | 2 | 2 | 2 |
| 2.5(S) | Dedicated budget available for PV-related activities | C | 0 | 0 | 0 | 0 | 0 | 0 |
| 2.8(S) | Existence of standard operating procedures (SOPs) for improving patient safety relating to medicine use | C | 2 | 2 | 0 | 2 | 2 | 2 |
| 2.9(S) | Existence of communication technologies to improve safety reporting and provision of medicine information | C | 2 | 2 | 0 | 2 | 2 | 2 |
| 2.10(S) | Existence of a medicine safety bulletin or newsletter published in the last six months | C | 0 | 0 | 0 | 0 | 0 | 0 |
| 2.11(S) | Existence of core reference materials available in the PV centre | S | 1 | 1 | 0 | 1 | 1 | 1 |
| 2.13(S) | Health care providers trained on pharmacovigilance and medicine safety in the last year | S | 0 | 0 | 0 | 1 | 1 | 1 |
|  | **Subtotal score (%)** |  | **11/18 (61)** | **11/18 (61)** | **0/18 (0)** | **12/18 (67)** | **12/18 (67)** | **12/18 (67)** |
|  | **III Signal generation and management** |  |  |  |  |  |  |  |
| 3.3 (P) | Existence of a form for reporting suspected ADRs | C | 2 | 2 | 2 | 2 | 2 | 2 |
| 3.4 (P) | Existence of a form for reporting suspected product quality issues | C | 2 | 2 | 2 | 2 | 2 | 2 |
| 3.5 (P) | Existence of a form for reporting suspected medication errors | C | 2 | 2 | 2 | 2 | 2 | 2 |
| 3.6 (P) | Existence of a form for reporting suspected treatment failure | C | 2 | 2 | 2 | 2 | 2 | 2 |
|  | **Subtotal score (%)** |  | **8/8 (100)** | **8/8 (100)** | **8/8 (100)** | **8/8 (100)** | **8/8 (100)** | **8/8(100)** |
|  | **IV Risk assessment and evaluation** |  |  |  |  |  |  |  |
| 4.1 (P) | Medicine utilization reviews carried out in the last year | S | 0 | 0 | 0 | 0 | 0 | 0 |
| 4.3 (P) | Quantification of the incidence of medication errors in the last year | S | 0 | 0 | 0 | 0 | 0 | 0 |
| 4.4 (P) | Number of ADR reports received in the last year | C | 0 | 0 | 0 | 2 | 0 | 0 |
| 4.5 (P) | Active surveillance activities currently ongoing or carried out in the last five years | C | 0 | 0 | 0 | 0 | 0 | 0 |
|  | **Subtotal score (%)** |  | **0/6 (0)** | **0/6 (0)** | **0/6 (0)** | **2/6 (33)** | **0/6 (0)** | **0/6 (0)** |
|  | **V. Risk management and communication** |  |  |  |  |  |  |  |
| 5.1 (O) | Risk mitigation plans currently in place that are targeted at high-risk medicines | S | 0 | 0 | 0 | 0 | 0 | 0 |
| 5.3 (O) | Number of medicine safety information requests received and addressed in the last year | S | 0 | 0 | 0 | 0 | 0 | 0 |
| 5.4 (O) | Planned issues of the medicine safety published in a newsletter or bulletin in the last year | S | 0 | 0 | 0 | 0 | 0 | 0 |
| 5.8 (O) | Percentage of the sampled drug and therapeutics committees that have carried out PV activities or addressed medicine safety issues in the last year | C | 0 | 0 | 0 | 0 | 0 | 0 |
|  | **Table 2 Result for Health facilities continued** |  |  |  |  |  |  |  |
|  | PV component | Type of indicator | CTH | PCMH | ODCH | BoGH | KeGH | MaGH |
|  |  |  |  |  |  |  |  |  |
|  | **Subtotal score (%)** |  | **0/5 (0)** | **0/5 (0)** | **0/5(0)** | **0/5 (0)** | **0/5 x100** | **0/5 x100** |
|  | **Total score achieved (%)** |  | **19/37** | **19/37** | **8/37** | **20/37** | **20/37** | **20/37** |
|  |  |  | **51%** | **51%** | **22%** | **54%** | **54%** | **54%** |

A grade of 2 points was allocated to each core indicator (C) fulﬁlled, 1 point for each supplementary indicator (S), and 0 points for any indicator not fulﬁlled. Thresholds set for quantitative indicators: 2.13 = 5 % of healthcare workers trained; 4.4 = 100 reports per million people; 5.3 = 100 per million people; 5.4 = at least 70 % of planned issues published; Connaught Teaching Hospital (CTH), Princess Christian Maternity Teaching Hospital (PCMH), Ola During Children Teaching Hospital (ODCH), Bo Government Hospital (BoGH), Kenema Government Hospital (KeGH) and Makeni Government Hospital (MaGH), outcome indicator (O), process indicator (P), structural indicator (S)

**Table S3: Result for the public health programmes**

|  | **Table 3 Result for public health programmes** |  |  |  |  |  |  |  |
| --- | --- | --- | --- | --- | --- | --- | --- | --- |
|  | PV component | Type of indicator | NMCP | NTDCP | EPI | HIV/AIDS | NLTCP | RCHP |
|  | **I Policy, Law and Regulation** |  |  |  |  |  |  |  |
| 1.1(S) | Policy document that contain essential elements on PV | C | 0 | 0 | 2 | 0 | 2 | 0 |
|  | Subtotal score (%) |  | **0/0 (0)** | **0/0 (0)** | **2/2 (100)** | **0/0 (0)** | **2/2 (100)** | **0/0(0)** |
|  | **II Systems, structures and stakeholders coordination** |  |  |  |  |  |  |  |
| 2.1(S) | Existence of a PV centre or unit | C | 2 | 2 | 2 | 2 | 2 | 2 |
| 2.2(S) | Clear mandate, structure, roles, and responsibilities for the PV centre | C | 0 | 0 | 0 | 0 | 0 | 0 |
| 2.4(S) | A designated staff responsible for PV | C | 2 | 2 | 2 | 2 | 2 | 2 |
| 2.5(S) | Dedicated budget available for PV-related activities | C | 0 | 0 | 0 | 0 | 0 | 0 |
| 2.8(S) | Existence of standard operating procedures (SOPs) for improving patient safety relating to medicine use | C | 0 | 0 | 2 | 0 | 0 | 0 |
| 2.9(S) | Existence of communication technologies to improve safety reporting and provision of medicine information | C | 0 | 0 | 0 | 0 | 0 | 0 |
| 2.13(S) | Health care providers trained on pharmacovigilance and medicine safety in the last year | S | 1 | 1 | 0 | 0 | 0 | 0 |
|  | **Subtotal score (%)** |  | **5/13 (38)** | **5/13 (38)** | **6/13 (46)** | **4/13 (31)** | **4/13 (31)** | **4/13 (31)** |
|  | **III Signal generation and management** |  |  |  |  |  |  |  |
| 3.3 (P) | Existence of a form for reporting suspected ADRs | C | 2 | 2 | 2 | 2 | 2 | 2 |
| 3.4 (P) | Existence of a form for reporting suspected product quality issues | C | 2 | 2 | 2 | 2 | 2 | 2 |
| 3.5 (P) | Existence of a form for reporting suspected medication errors | C | 2 | 2 | 2 | 2 | 2 | 2 |
| 3.6 (P) | Existence of a form for reporting suspected treatment failure | C | 2 | 2 | 2 | 2 | 2 | 2 |
|  | **Subtotal score (%)** |  | **8/8 (100)** | **8/8 (100)** | **8/8 (100)** | **8/8 (100)** | **8/8 (100)** | **8/8(100)** |
|  | **IV Risk assessment and evaluation** |  |  |  |  |  |  |  |
| 4.1 (P) | Medicine utilization reviews carried out in the last year | S | 0 | 0 | 0 | 0 | 0 | 0 |
| 4.2 (P) | Pharmaceutical quality survey conducted in the last 5 years | S | 1 | 0 | 0 | 1 | 0 | 0 |
| 4.3 (P) | Quantification of the incidence of medication errors in the last year | S | 1 | 0 | 0 | 0 | 0 | 0 |
| 4.4 (P) | Number of ADR reports received in the last year | C | 2 | 2 | 0 | 0 | 0 | 0 |
| 4.5 (P) | Active surveillance activities currently ongoing or carried out in the last five years | C | 0 | 0 | 0 | 0 | 0 | 0 |
| 4.6 (P) | Percentage of patients in public health programs for whom drug-related adverse events were reported in the last year (disaggregated by type of adverse event, drug, severity, outcomes, and demographics) | C | 2 | 2 | 0 | 0 | 0 | 0 |
| 4.7 (P) | Percentage of patients undergoing treatment within a public health program whose treatment was modified because of treatment failure or ADRs in the last year (disaggregated by treatment failure and ADRs) | C | 0 | 0 | 0 | 0 | 0 | 0 |
| 4.8 (P) | Percentage of patients in public health programs for whom drug-related, serious “unexpected adverse events” were reported in the last year | S | 1 | 1 | 0 | 0 | 0 | 0 |
|  | **Subtotal score (%)** |  | **7/12 (58)** | **5/12 (42)** | **0/12 (0)** | **1/12 (8)** | **0/12 (0)** | **0/12 (0)** |
|  | **Table 3 Result for public health programmes continued** |  |  |  |  |  |  |  |
|  | PV component | Type of indicator | NMCP | NTDCP | EPI | HIV/AIDS | NLTCP | RCHP |
|  | **V. Risk management and communication** |  |  |  |  |  |  |  |
| 5.1 (O) | Risk mitigation plans currently in place that are targeted at high-risk medicines | S | 1 | 1 | 1 | 1 | 1 | 1 |
| 5.2 (O) | Prequalification schemes used in medicine procurement decisions | S | 1 | 1 | 1 | 1 | 1 | 1 |
| 5.3 (O) | Number of medicine safety information requests received and addressed in the last year | S | 0 | 0 | 0 | 0 | 0 | 0 |
| 5.7 (O) | Average time lag between identification of safety signal of a serious ADR or significant medicine safety issue and communication to health care workers and the public | C | 0 | 0 | 0 | 0 | 0 | 0 |
| 5.9 (O) | Public or community education activities relating to medicine safety carried out in the last year | S | 1 | 1 | 0 | 0 | 0 | 0 |
|  | **Subtotal score (%)** |  | **3/6 (50)** | **3/6 (50)** | **2/6(33)** | **2/6(33)** | **2/6(33)** | **2/6(33)** |
|  | **Total score achieved (%)** |  | **22/41** | **21/41** | **18/41** | **15/41** | **16/41** | **14/41** |
|  |  |  | **54%** | **51%** | **44%** | **37%** | **39%** | **34%** |

A grade of 2 points was allocated to each core indicator (C) fulﬁlled, 1 point for each supplementary indicator (S), and 0 points for any indicator not fulﬁlled. Thresholds set for quantitative indicators: 2.13 = 5 % of healthcare workers trained; 4.4 = 100 reports per million people; 5.3 = 100 per million people; 5.4 = at least 70 % of planned issues published National Malaria Control Programme (NMCP), Neglected Tropical Disease Control Programme (NTDCP), Expanded Programme for Immunisation (EPI), HIV/AIDS Control Programme, National Leprosy and Tuberculosis Control Programme(NLTCP), Reproductive and Child Health Programme(RCHP), Outcome indicator (O), process indicator (P), structural indicator (S).
